# Attitudes of nurses toward life-sustaining treatment and its influencing factors: A mixed-methods study

**DOI:** 10.1016/j.ijnss.2026.08.008

**Published:** 2026-08-14

**Authors:** Hyebin Oh, Ju-Young Ha

**Affiliations:** a College of Nursing, Pusan National University, Yangsan, the Republic of Korea; b Department of Nursing, Pusan National University Hospital, Busan, the Republic of Korea

**Keywords:** Attitude to death, Life support care, Nurses, The Republic of Korea, Terminal care

## Abstract

**Objective:**

This study aimed to identify factors influencing nurses’ attitudes toward life-sustaining treatment (LST) in the Republic of Korea and to explore how perceptions of a good death and contextual factors shape these attitudes.

**Methods:**

An explanatory sequential mixed-methods study was conducted at two tertiary hospitals from July to November 2024. In the quantitative phase, 104 nurses completed a cross-sectional survey, and multiple linear regression was used to identify factors associated with LST attitudes. In the qualitative phase, eight purposively sampled nurses participated in semi-structured interviews, which were analyzed using conventional content analysis. Findings were integrated through a joint display.

**Results:**

Quantitative findings showed moderate good death perceptions (mean = 2.91) and negative LST attitudes (mean = 3.52). Male gender (*β* = 0.32, *P* < 0.001) and stronger good death perceptions (*β* = 0.26, *P* = 0.005) were associated with more negative LST attitudes. Qualitative findings identified seven domains: experiences of close others’ deaths; direct patient care experience; depth of LST knowledge; patient age, prognosis and recovery potential; family economic burden; traditional Korean cultural values; social acceptance and legal liability concerns. Integrated findings showed that traditional cultural values and gender roles contextualized the association between gender and gender roles. At the same time, experiences of death and end-of-life care explained the association between good death perceptions and LST attitudes.

**Conclusion:**

Nurses’ LST attitudes are shaped by good death perceptions as well as personal, clinical, and sociocultural factors. These findings provide an empirical basis for developing competency-based LST education, standardized case-based protocols, and culturally sensitive practice guidelines to support nurses in end-of-life decision-making.

## What is known?

• Nurses play a critical role as mediators and counselors in life-sustaining treatment (LST) decision-making.
• Previous research has primarily relied on quantitative surveys, which are often limited to ICU settings.

## What is new?

• This study employed an explanatory sequential mixed-methods design to contextualize how sociocultural factors shape quantitative predictors (gender and perceptions).
• It identifies traditional Korean values, such as filial piety and patriarchal decision-making, as key mechanisms explaining gender differences in LST attitudes.
• The findings clarify that personal and clinical encounters with “futile” treatments ultimately solidify a nurse’s stance against LST continuation.

## 1 Introduction

Globally, population aging and advances in medical technology have increased the complexity of decisions regarding life-sustaining treatment (LST), prompting greater emphasis on patients’ autonomy, a dignified death, and advance care planning [1,2]. Since the Act on Decisions on LST was enacted in the Republic of Korea in 2016, public participation emphasizing self-determination has steadily grown and reshaped clinical practice. In 2023, national registrations of advance directives for LST reached 573,937, an increase of 39.4 % from 2022. Completion of LST planning forms totaled 25,197, representing a 7.1 % increase from the previous year, and documented decisions to withdraw or withhold LST numbered 70,720, marking a 10.6 % increase [3].

In 2023, 75.4 % of all deaths in the Republic of Korea occurred in healthcare institutions [4]. During inpatient LST decision-making, healthcare professionals, especially nurses, who communicate closely with patients and their surrogates, play an important role [5]. Nurses provide accurate information, act as counselors and facilitators, and are expected to participate actively in LST-related decision-making [6,7].

Nurses’ LST attitudes can shape their interventions and influence decisions made by patients and families [8]. Nurses’ attitudes affect their effectiveness as counselors, their ability to support informed decision-making [9], and the overall quality of care provided [10,11]. At the same time, nurses often face internal conflict between their duty to preserve life and their ethical responsibility to relieve suffering and respect patients’ right to a dignified death [12]. When nurses feel unable to ensure a peaceful, dignified, pain-free death for their patients, they often report moral distress, regret, and anxiety [13]. For this reason, nurses caring for dying patients must reflect on their LST attitudes and establish consistent values and standards to support patients and families in making better decisions and delivering nursing care while enhancing quality of life near the end of life.

Multiple factors influence nurses’ LST attitudes, including awareness of biomedical ethics, religion, age, clinical unit, perceptions of a good death, gender, knowledge of advance directives, and education on the “good-death” concept [10,[14], [15], [16]]. Among these, the perception of a good death is a particularly strong predictor [10,16]. In the Republic of Korea, a good death is understood as a collaborative, continuous process in which patients, families, and healthcare providers strive to fulfill end-of-life wishes. This approach allows patients to die with dignity and self-determination, while helping families to experience positive emotions [17]. Nurses with more positive good death perceptions are better able to provide high-quality end-of-life care [17], display more positive attitudes toward end-of-life care [18], and influence LST-related decisions [19]. Positive perceptions of death may reduce patients’ fear of dying and promote peaceful death in a safe and respectful environment [20]. Conversely, negative or underdeveloped perceptions heighten anxiety, leading to psychological burdens such as fear, depression, and stress [18]. These challenges may cause nurses to avoid discussing death or to rely on defense mechanisms [21], ultimately reducing care quality and undermining patients’ quality of life. As the meaning of a good death varies across sociocultural contexts and personal values, and may have shifted following the introduction of the LST decision-making system [22], it is necessary to explore in depth how clinical nurses conceptualize a good death and how these perceptions shape their LST attitudes.

Most studies have relied on quantitative surveys of ICU or general hospital nurses, and few have integrated perceptions of death and LST attitudes [10,[14], [15], [16]]. Given that perceptions of death are subjective and shaped by culture and experience, questionnaires alone may not capture their full scope. Mixed-method approaches can offer a more comprehensive understanding [23]. Accordingly, this study used a quantitative survey to assess nurses’ perceptions of a good death, their LST attitudes, and the relationship between these perceptions and attitudes. In-depth interviews were conducted to explore individual experiences, values, and emotions related to death and LST attitudes. As nurses in emergency and oncology units frequently encounter dying patients [24], participants were recruited from these settings. Ultimately, this study aimed to identify the factors influencing nurses’ LST attitudes in the Republic of Korea and to provide a deeper understanding of how personal and sociocultural contexts shape these factors through an explanatory sequential mixed-methods design. These findings provide foundational evidence to support nurses’ roles in LST decision-making and promote high-quality end-of-life nursing care.

## 2 Methods

### 2.1 Study design

This study used an explanatory sequential mixed-methods design. First, a descriptive survey was conducted to assess nurses’ good death perceptions and their attitudes toward LST. Based on the survey findings, in-depth interviews were conducted to contextualize the quantitative results. This study was reported in accordance with the Good Reporting of a Mixed Methods Study (GRAMMS) guidelines [25] to ensure methodological transparency and rigorous reporting in the mixed-methods design.

### 2.2 Quantitative research

#### 2.2.1 Study design and participants

This cross-sectional study was conducted from July 1 to September 15, 2024. Eligible participants included registered nurses who had cared for at least one dying patient in the ICU, emergency department (ED), hospice ward, or oncology ward at two tertiary hospitals: Pusan National University Hospital (Busan Metropolitan City) and Pusan National University Yangsan Hospital (Gyeongsangnam-do Province) in the Republic of Korea. We excluded nurses working in operating rooms, post-anesthesia care units, and outpatient settings, as well as those with fewer than 10 months of clinical experience.

#### 2.2.2 Sample size calculation

The minimum required sample size was estimated using G∗Power version 3.1.9.7, based on an *F*-test for multiple linear regression with 17 predictors [26]. The calculation targeted the overall model effect, assuming a medium effect size (*f*^2^ = 0.30), a two-sided significance level (*α*) of 0.05, and a statistical power (1-*β*) of 0.90. Under these assumptions, the minimum sample size was estimated to be 97 participants. Considering a 10 % attrition rate, the target sample size was set at 108 participants. In total,104 participants were included in the final analysis.

#### 2.2.3 Measures

##### 2.2.3.1 Demographic and clinical characteristics

Data were collected for the following variables: gender, age, marital status, religion, education level, total clinical experience, current unit and tenure, position, experience with a family member’s or acquaintance’s death within the past year, prior and past year LST nursing care (including withdrawal/withholding), etc.

##### 2.2.3.2 Perception of a good death

Perceptions were assessed using the official Korean translation of the Measure of Concept of a Good Death, which was originally developed and registered by Schwartz et al. [27]. Formal authorization to utilize this instrument was obtained through a commercial licensing agreement with the DeltaQuest Foundation, Inc., which holds the proprietary rights to the validated Korean version. The instrument consists of 17 items across three subdomains: clinical symptoms (5 items), intimacy (9 items), and sense of control (3 items). Items are rated on a 4-point Likert scale (1 = not at all necessary to 4 = absolutely necessary), with higher scores reflecting more positive perceptions. For statistical analysis, the scores were calculated as an item-level mean score (ranging from 1 to 4) by dividing the total summated score by the number of items (17). Cronbach’s α coefficient was 0.87 in the original study [27] and 0.84 in this study.

##### 2.2.3.3 Attitudes toward life-sustaining treatment

LST attitudes were measured using a 17-item instrument developed by Lee and Kim [7], originally adapted from Park [28] and revised by Byun et al. [29]. This version had been pre-modified to align with the Act on Decisions on LST by excluding two legislatively inconsistent items and operationalizing “irreversible patients” as “patients receiving LST”. Formal permission to use this final version was obtained from the author before data collection. No further modifications or revisions were made to the questionnaire during our survey process. The scale consists of three pro-LST continuation items(reverse-scored) and 14 items supporting LST withdrawal or withholding. Each item is scored on a 5-point Likert scale (1 = strongly disagree to 5 = strongly agree), and individual item scores after reverse-coding were calculated as an item-level mean score (ranging from 1 to 5). Higher mean scores indicate more negative attitudes toward the continuation of LST. Cronbach’s α coefficient was 0.78 in Lee and Kim’s study [7] and 0.80 in this study.

#### 2.2.4 Data collection and quality control

The nursing administrations of both hospitals granted permission. Recruitment notices outlining the purpose, procedures, and ethical safeguards of the study were posted at nursing stations in the ED, ICU, hospice ward, and oncology ward. Nurses who voluntarily consented to participate completed paper questionnaires, sealed them in opaque envelopes, and returned them directly to the investigator.

#### 2.2.5 Data analysis

Data were analyzed using SPSS v28. Categorical variables are expressed as frequencies and percentages. Normally distributed variables are presented as mean and standard deviation (SD), while non-normally distributed variables are summarized as Median (*P*_25_, *P*_75_). To evaluate group differences, non-parametric tests-specifically, the Mann–Whitney *U* test or Kruskal–Wallis *H* test-were applied due to small and unbalanced sample sizes in some subgroups. Pearson’s correlation coefficient was used to examine the associations between good-death perceptions and LST attitudes. Multiple linear regression was used to identify factors associated with LST attitudes, with the mean score across 17 five-point Likert items treated as a continuous dependent variable, a practice justified by simulation evidence supporting parametric analysis of Likert‑derived composite scores with five or more response options [30]. Regression assumptions were assessed and met.

### 2.3 Qualitative research

#### 2.3.1 Study participants

For the qualitative phase, eight nurses were purposively sampled, comprising four volunteers from the initial survey cohort and four additional recruits via nursing station posts. All survey participants were asked about their willingness to participate in interviews; the four who agreed were subsequently contacted via text message to confirm. Male nurses were specifically targeted to explore further the gender-related differences identified in the quantitative analysis. Given the high specificity of the participants’ clinical expertise, this sample size provided sufficient “information power” [31] to achieve robust analytical depth. Consequently, recruitment concluded once data saturation was reached, as the final two interviews yielded no new themes.

#### 2.3.2 Interview outline

A semi-structured interview guide, informed by quantitative findings (e.g., gender differences and perceptions of good death), included the following questions. 1) How do you define and experience a good death? 2) What thoughts and emotions shape your views on LST? 3) What is the nurse’s role in withdrawal or withholding decisions? 4) Have you observed gender-related differences in emotions or LST attitudes? 5) How do good death perceptions influence your decision-making and nursing care?

#### 2.3.3 Data collection and quality control

Following the quantitative analysis, qualitative data were collected from October 15 to November 20, 2024. Before each interview, the investigator explained the study’s purpose, procedures, and right to withdraw and obtained written informed consent. Individual interviews (30–60 min) were conducted in private spaces chosen by the participants and audio-recorded. If clarification was needed, follow-up phone calls (≤20 min) were conducted. Data collection continued until both researchers agreed that no new codes or subthemes had emerged from the interviews [32].

#### 2.3.4 Data analysis

Qualitative data were analyzed inductively using the conventional content analysis methods of Elo and Kyngäs [33]. This inductive approach enabled a nuanced exploration of nurses’ unique perspectives and experiences without the constraints of preconceived categories, aligning with the study’s focus on uncovering complex influencing factors. Each interview was transcribed verbatim and reviewed repeatedly to achieve immersion. Following this systematic framework, the analysis proceeded iteratively through open coding, categorization, and abstraction, ensuring a transparent and rigorous examination of the participants’ narratives.

To ensure rigor, we applied Lincoln and Guba’s [34] criteria. 1) Credibility: The data were independently analyzed by two researchers (a master’s student who received intensive training in qualitative content analysis and a nursing professor [PhD] with extensive expertise in qualitative nursing research). 2) Transferability: Participants were purposively selected based on their extensive experience with end-of-life care. 3) Dependability: Inductive analysis was conducted according to Elo and Kyngäs’s methodology with all documented steps. 4) Confirmability: To maintain objectivity and minimize individual bias, researchers maintained reflexive journals throughout the study period.

### 2.4 Mixed research

This study used a sequential explanatory mixed-methods design, in which the initial quantitative findings (specifically, gender-related differences and perceptions of a good death) directly informed the development of the qualitative semi-structured interview guide. Furthermore, data integration occurred at the interpretation level through a joint display matrix during the discussion phase. The qualitative narrative insights were embedded to expand, clarify, and contextualize the statistical results, thereby contextualizing the factors influencing nurses’ LST attitudes.

### 2.5 Ethical consideration

This study was approved on May 23, 2024, by the Institutional Review Board of Pusan National University (No. 2024_68_HR). All participants provided written informed consent after receiving detailed explanations of the study. Participation was voluntary, and nurses were informed of their right to withdraw without penalties. Upon completion of the interviews, participants received a modest token of appreciation.

## 3 Results

### 3.1 Participant characteristics

The participants’ characteristics are summarized in Appendix A. Of the 104 survey respondents, the mean age was 32.0 ± 4.2 years. More than half were single (51.9 %), two-thirds were non-religious (66.3 %), and 67.3 % held a bachelor’s degree. Mean clinical experience was 8.18 ± 4.22 years (with a mean current department experience of 4.79 ± 3.55 years), and 34.6 % worked in the ICU. Nearly all the participants (99.0 %) were staff nurses. More than half (54.8 %) of the participants had received no LST-related education in the past year, and 43.3 % “sometimes” thought about a good death. To complement the survey demographics and gain a deeper contextual understanding, the qualitative interviews involved eight nurses (four women and four men) with a mean age of 30.9 years and a mean experience of 6.5 years. To ensure diverse clinical perspectives, participants were recruited from multiple settings: one hospice care setting, four EDs, and three ICUs (Appendix B).

### 3.2 Results of the quantitative survey

#### 3.2.1 Good death perceptions

Overall, good death perceptions were moderate (2.91 ± 0.39), with the intimacy subdomain scoring the highest (Appendix C). The top-rated item was “providing the patient with an opportunity to bid farewell” (mean = 3.63), highlighting the value of emotional closure. Pain-free death was also prioritized (“absence of pain or being largely pain-free”, mean = 3.56). The lowest-rated item was “sudden and unexpected” (mean = 1.53). Finally, a significant difference was observed in perceptions among groups with different levels of total clinical experience (*H* = 6.71, *P* = 0.035) (Appendix A).

#### 3.2.2 Life-sustaining treatment attitudes

The composite mean attitude score for LST was 3.52 ± 0.48, reflecting a negative stance (Appendix D). The highest-rated item was “Patients have the right to decide about their own death” (mean = 4.67). Significant gender differences were found in the quantitative results (*Z* = −2.14, *P* = 0.032).

#### 3.2.3 Factors influencing life-sustaining treatment attitudes

Multiple regression identified gender (*β* = 0.32, *P* < 0.001) and perceptions of a good death (*β* = 0.26, *P* = 0.005) as independent predictors of negative attitudes toward LST continuation (Appendix E). The model was significant (*F* = 10.44, *P* < 0.001) and explained 16 % of the variance (*R*^2^ = 0.16).

### 3.3 Results of the qualitative interview

#### 3.3.1 Good death perceptions

The interviews elaborated on the good death perceptions, with participants emphasizing the importance of having time to prepare for the end of their lives:

*“I do not necessarily view cancer as a bad way of dying. Given a choice, I’d prefer cancer over a sudden death from an accident, because with cancer you can anticipate it to some extent—you have time to say yes and to put your affairs in order.”* (Participant 1)

Mirroring the quantitative finding that a pain-free death is a priority, nurses emphasized that minimizing suffering and preserving dignity were core elements of a good death:

*“The most important thing is to die without pain—whether* via *morphine or other analgesics–and effective pain control is paramount.”* (Participant 5)

Participants also described sudden and unexpected deaths as psychologically burdensome for families:

*“My friend’s father died abruptly from trauma, and his family had no time to prepare. In contrast, our father’s cancer, regardless of the disease course, allowed us to contemplate mortality, think ‘this could happen,’ and emotionally prepare for his passing. Without this time, it would have been much more painful.”* (Participant 3)

#### 3.3.2 Life-sustaining treatment attitudes

Mirroring this focus on autonomy, the interviews strongly supported patients’ right to choose:

*“I’ve cared for patients who said, ‘I can’t do this anymore; I want to stop,’ yet their families urged, ‘Let’s keep trying; there’s hope.’ In these cases, I prioritized the patient’s wishes and sought further discussions with the family. It is the patient’s decision, not ours or their families, to make.”* (Participant 5)

The qualitative data provided a deeper cultural explanation for these attitudes, specifically through the lens of traditional Korean decision-making norms.

*“Because men still often bear financial and practical decision-making responsibilities in the family, they tend to feel the burden and make sooner, more pragmatic decisions in LST situations than women. The eldest son or a male family member frequently makes important choices.”* (Participant 6)

#### 3.3.3 Contextual descriptions of factors influencing life-sustaining treatment attitudes

To complement the quantitative findings, the qualitative findings provided contextual descriptions of how these factors and other influences operate in clinical practice, organized into three domains.

##### 3.3.3.1 Personal factors

(1) Experiences of close others deaths

Participants who had witnessed the death of a family member or close friend reported more positive LST attitudes and withdrawal. The personal narratives illustrate the specific life experiences that contribute to the “good death perceptions,” which was identified as a significant predictor in the regression model:

*“My attitude toward LST was shaped by my father’s 10-year battle with metastatic cancer, during which he endured profound suffering and pain from chemotherapy and surgery. When he expressed a wish to stop treatment, our family completed the withdrawal forms, and he died peacefully in the hospice. Observing my grandmother’s dialysis and decline also helped prepare him. From both experiences, I came to view LST withdrawal as appropriate and necessary.”* (Participant 3)

(2) Direct patient care exposure

Nurses with extensive clinical tenure who repeatedly witnessed futile interventions shifted toward viewing withdrawal as compassionate.

*“Early in my career, I felt indebted as if I had to ‘do everything’ for a patient, just as I would for my own parents. I assumed this was the right thing to do. Over time, however, seeing patients after aggressive Cardiopulmonary Resuscitation—covered in blood, intubated, and hooked to multiple devices—made me realize they would not have wanted that.”* (Participant 5)

(3) Depth of life-sustaining treatment knowledge

Greater familiarity with the Act on Decisions on LST fostered advocacy for the withdrawal of LST and informed discussions with families. The interviews identified LST knowledge as a practical factor that facilitated the translation of nurses’ attitudes toward clinical advocacy.

*“When I wrote a paper on a similar topic before the Act was enacted, I closely followed the system’s development and thought it was an excellent and necessary measure. Even now, when a patient’s condition worsens, I inform them or their surrogates, ‘There is such a system—please consider it in advance.’ I often ask the attending physicians to initiate that conversation.”* (Participant 8)

##### 3.3.3.2 Patient and surrogate factors

(1) Patient age, prognosis, and recovery potential

Younger patients were viewed as more likely to benefit from LST, while older patients with poor prognosis were seen as suffering needlessly. These narratives describe the specific clinical criteria that nurses consider when deliberating on the need for LST beyond the variables measured in the survey:

*“My feelings change depending on age. Those in their 20–30s still have most of their life ahead; I feel more compelled to ‘give it a try’ for younger patients …”* (Participant 7)

*“Ventilator weaning succeeds frequently in 40–50-year-olds—they’re still young—so I want to extend LST for them*.*”* (Participant 8)

*“When deciding on LST, the key factor I consider is the likelihood of returning to normal life … if they remain machine-dependent and unable to communicate, continuing LST is questionable*.*”* (Participant 5)

(2) Family economic burden

The high costs of end-of-life care influenced participants’ LST attitudes and decisions. The participants explained that surviving family members had to carry the financial burden. The interviews highlighted financial realities as a practical factor explaining the complexities of surrogate decision-making.

*“End-of-life care incurs the highest costs, and modalities like Continuous Renal Replacement Therapy or Extracorporeal Membrane Oxygenation can generate unexpectedly large bills. Surrogates often request ‘everything’ without knowing the expense; whereas physicians may proceed, we inform families, ‘This intervention could cost X—please consider it.’”* (Participant 8)

##### 3.3.3.3 Social factors

(1) Traditional Korean values

Participants reported that Confucian values are deeply rooted in Korean society, shaping LST attitudes and often leading to the view that withdrawal is unfilial. The interviews elaborated on how these cultural values influenced LST attitudes and the decision-making hierarchy within families.

*“Confucian ideals persist and many believe that not pursuing every possible treatment for parents constitutes filial neglect. Consequently, older surrogates often demand full LST, regardless of prognosis, whereas younger surrogates are more willing to limit treatment when they seem futile.”* (Participant 4)

*“Despite societal shifts, economic authority still resides with the male head of household, who traditionally manages funeral arrangements and thus makes pragmatic end-of-life choices.”* (Participant 6)

(2) Social acceptance and legal liability concerns

The qualitative narratives explain the discrepancy between nurses’ professional attitudes and their actual clinical practice, identifying external pressures, such as legal liability and social norms, as significant barriers to implementing LST withdrawal.

*“In practice, many patients and surrogates still do not know about this system, and I have observed that physicians avoid stopping treatment to sidestep responsibility. Even when families complete withdrawal forms and request extubation, clinicians often say, ‘Well, the ventilator is already in place, let us just keep it going.’ They worry about the potential legal issues if something goes wrong later in life.”* (Participant 3)

### 3.4 Results of mixed studies

To synthesize nurses’ LST attitudes, findings from the quantitative and qualitative phases were integrated using a joint display (Table 1). Qualitative data provide interpretive depth for quantitative findings. Specifically, the patriarchal decision-making structure and traditional values in the Republic of Korea explain why male nurses hold more pragmatic and conservative LST attitudes. Furthermore, the qualitative phase expanded the survey results by identifying critical factors not captured in the quantitative instruments, such as the psychological impact of patient age, practical legal liability concerns, and the family’s economic burden. These integrated insights demonstrate that nurses’ LST attitudes may be shaped by individual perceptions of a “good death” and by a complex interplay of clinical experiences and socio-cultural barriers.

**Table 1:** Joint display of quantitative predictors and qualitative explanations.

Quantitative results	Qualitative themes	Integrated insights
Male gender (*β* = 0.32, *P* < 0.001)	Traditional Korean values and patriarchal decision-making	Traditional patriarchal values and gendered roles in the Republic of Korea influence male nurses to adopt more pragmatic and conservative attitudes toward LST continuation.
Good death (*β* = 0.26, *P* = 0.005)	Dignified end-of-life and pain management	Nurses believe futile LST undermines the value of a “good death”; thus, stronger perceptions of dignity and pain-free death lead to higher opposition toward LST.
Not significant or not measured in the quantitative phase	Depth of LST knowledge and legal proficiency	Practical legal knowledge, rather than more formal education, serves as a key driver for professional advocacy and counseling in clinical LST decisions.
	Patient age, prognosis, and recovery potential	Patient age acts as a psychological criterion beyond statistical measures; younger patients are prioritized for treatment based on “hope” and recovery potential.
	Family economic burden and social support	LST attitudes are shaped not only by personal values but also by the family’s economic capacity and the practical reality of surrogate decision-making.
	Social acceptance and legal liability concerns	Anxiety regarding legal liability and limited social acceptance creates a gap between nurses’ professional attitudes and actual clinical practice.

*Note*: LST = life sustaining treatment.

## 4 Discussion

This mixed-methods study examined how tertiary hospital nurses’ perceptions of a good death influence their LST attitudes. Quantitative findings showed that both male gender and stronger good-death perceptions predicted more negative LST attitudes. Qualitative interviews revealed additional personal, patient, and sociocultural influences. Integrating these phases provided a more comprehensive understanding of the determinants of nurses’ LST attitudes.

Nurses reported moderate good death perceptions, with intimacy being valued the most, followed by symptom control and autonomy. These findings align with prior studies [18], showing that “time to say goodbye” and a “comfortable, pain-free death” are central to a peaceful death. Participants stressed that pain relief preserves dignity and minimizes suffering, underscoring its importance as a core nursing role in end-of-life care [35].

Nurses’ overall LST attitudes were negative. This score is higher than those reported in ICU-only samples [6,7], likely reflecting contributions from emergency, oncology, and hospice nurses, who encounter broader end-of-life contexts and often receive additional training [36,37]. Attitudes consistently emphasize patient autonomy, and both surveys and interviews highlight that respecting patients' expressed wishes is paramount. This finding is further supported by a recent cross-sectional study conducted in China, which reported that greater patient participation in end-of-life decision-making and more extensive communication with healthcare providers regarding illness, treatment, and death-related issues were associated with a higher quality of dying and death among patients with terminal cancer [38]. Together, these findings underscore the importance of respecting patients’ preferences and promoting shared decision-making in end-of-life care.

In this study, male nurses expressed more negative LST attitudes than female nurses, consistent with prior ICU research [6,16]. Interviews linked this to traditional Korean norms that assign primary decision-making authority and financial responsibility to men [39], thereby shaping more pragmatic and cost-conscious views of LST. Given the small number of male participants, larger and more diverse samples are needed to clarify gender effects.

Positive perceptions of death as a good also predicted negative LST attitudes, consistent with prior nurse-focused research [10,16]. Nurses who valued farewells and pain-free deaths saw LST as prolonging suffering, preventing closure, and compromising dignity [40]. Interviews confirmed that cardiopulmonary resuscitation and mechanical ventilation often intensified physical distress and prevented meaningful final moments, reinforcing nurses’ opposition to LST continuation.

The qualitative interviews revealed seven additional influences. Nurses’ personal experiences of the deaths of close others, especially those marked by futile interventions or unnecessary suffering, deepened their opposition to aggressive treatment. Clinical exposure plays a similar role, with repeated encounters with futile LST shifting views toward valuing comfort and dignity, as described by Yun et al. [41] and others [42,43]. Veteran nurses, especially those practicing before the enactment of the LST Act, reported that aggressive life-sustaining measures often prolonged suffering, compromised dignity, and psychologically and financially burdened families. These cumulative encounters, rather than the sheer length of service, may shape nurses’ perspectives on LST.

Knowledge of the Act on Decisions on LST strengthened nurses’ opposition to unwarranted treatment, aligning with prior studies that link legal literacy to treatment withdrawal [16,44,45]. Knowledge of this legislation may help nurses navigate end-of-life legal and ethical dilemmas and facilitate patient-centered decision-making [44]. Prior studies have demonstrated that ongoing comprehensive LST education enhances nurses’ legal competence and the quality of end-of-life care [16]. This supports calls for structured experiential curricula beyond didactic sessions.

Patient age and prognosis were central to shaping nurses’ LST attitudes. Participants supported LST for younger patients with recovery potential but were less supportive of LST for older patients or those with poor prognoses, where treatment was seen as prolonging suffering. Similarly, Lee [46] reported that nurses held negative attitudes toward life-prolonging measures that offered no therapeutic benefit.

Nurses’ LST attitudes were also shaped by the economic and emotional burdens borne by surviving family members. Families often incur substantial financial strain from repeated procedures and experience escalating emotional distress and regret as treatment is prolonged [39]. Nurses, having witnessed these cumulative burdens first-hand, felt responsible for facilitating LST decisions that consider the patient’s prognosis and the family's overall circumstances. Thus, nurses must support end-of-life decision-making by guiding families in weighing patient benefits against the economic and emotional tolls, thereby facilitating informed, regret-minimized choices.

Cultural values also influenced attitudes. Confucian traditions frame withdrawal as unfilial, while patriarchal norms give male relatives authority over their decisions. Cultural perceptions of good death vary. Western societies emphasize individual autonomy, whereas Asian cultures prioritize family-centered, intergenerational decision-making [22]. Older surrogates often demand maximal treatment, while younger ones leaned toward withdrawal of LST, reflecting shifting generational perspectives [22]. These findings highlight the interplay between autonomy, family-centered decision-making, and evolving social norms in the Republic of Korea.

Despite the support for the LST Act, barriers remain. Interviews revealed that limited public awareness, ambiguous legal language, and clinicians’ liability concerns deter clinicians from withdrawing LST. Although patient and surrogate awareness has modestly increased since enactment [22], the understanding of withdrawal options remains inadequate. Clinicians report confusion over complex legal requirements and vague distinctions between “terminal” and “dying” phases, undermining effective counseling and proper application of the law [47,48]. Physicians often continued treatment even when withdrawal forms were completed, reflecting low system literacy and procedural uncertainty. Addressing these barriers requires clearer guidelines, broader public education, and ongoing hands-on training for healthcare professionals.

## 5 Limitations

First, while data saturation and sufficient information power were achieved, the qualitative phase was conducted with a relatively homogeneous group of eight participants from similar clinical environments across two tertiary hospitals. This institutional homogeneity may have constrained the diversity of the lived experiences captured, potentially limiting the transferability of the findings to nurses in other healthcare settings, such as secondary or long-term care hospitals, where LST protocols and resources differ markedly. Second, the participants’ relatively young age may not capture diverse generational perspectives or varying levels of clinical maturity in end-of-life care. In addition, the highly skewed distribution of participants across certain demographic and clinical variables required the use of non-parametric analyses for group comparisons, which may limit the generalizability of the findings. Future research should involve larger multicenter cohorts stratified by age, sex, and years of clinical experience to ensure a more comprehensive representation. Further investigation into organizational and sociocultural variables is warranted to generate broader insights into evidence-based LST decision-making in diverse nursing practices.

## 6 Conclusions

In this study, nurses defined a good death as one characterized by emotional connection and effective pain management. Male and stronger good-death perceptions predicted more negative LST attitudes. The qualitative findings highlighted the role of personal experiences, clinical exposure, prognosis, family burden, and sociocultural norms. To support nurses in LST decision-making, competency-based training on the LST Act, standardized case protocols, and clear clinical guidelines are essential. Broader, multisite studies should further examine organizational and cultural determinants to strengthen patient-centered and ethically sound end-of-life care.

## Data availability statement

The datasets generated during and/or analyzed during the current study are available from the corresponding author upon reasonable request.

## CRediT authorship contribution statement

**Hyebin Oh:** Conceptualization, Methodology, Software, Formal analysis, Investigation, Resources, Data curation, Writing – original draft, Writing – review & editing, Visualization, Project administration. **Ju-Young Ha:** Conceptualization, Methodology, Validation, Formal analysis, Resources, Writing – review & editing, Supervision, Project administration.

## Declaration of competing interest

The authors have declared no conflict of interest.
