# Supplementary material for: Attitudes of nurses toward life-sustaining treatment and its influencing factors: A mixed-methods study
Source: Int J Nurs Sci. 2026 Aug 14;13(5):459–66. doi: 10.1016/j.ijnss.2026.08.008 (PMC13648181; doi:10.1016/j.ijnss.2026.08.008)
Supplement: Supplementary file 1 [file mmc1.docx]

Appendix A. Good death recognition and attitude toward life-sustaining treatment according to participants’ general characteristics(*n* = 104)

| Characteristics | *N* (%) | Good death recognition | | | Attitude toward life-sustaining treatment | | |
| --- | --- | --- | --- | --- | --- | --- | --- |
|  |  | *Median* (*P*_25_*, P*_75_) | *Z/H* | *P* | *Median* (*P_25_, P*_75_) | *Z/H* | *P* |
| Gender |  |  | ﹣0.78 ^a^ | 0.436 |  | ﹣2.14 ^a^ | 0.032 |
| Male | 4 (3.8) | 2.56 (2.53, 2.93) |  |  | 4.32 (3.78, 4.82) |  |  |
| Female | 100 (96.2) | 2.94 (2.65, 3.18) |  |  | 3.53 (3.18, 3.78) |  |  |
| Age (year) |  |  |  |  |  |  |  |
| 20–29 | 29 (27.9) | 2.82 (2.65, 3.12) | 0.29 ^b^ | 0.864 | 3.53 (3.18, 3.76) | 1.69 ^b^ | 0.429 |
| 30–39 | 71 (68.3) | 2.94 (2.59, 3.18) |  |  | 3.47 (3.21, 3.82) |  |  |
| 40–49 | 4 (3.8) | 2.94 (2.68, 3.28) |  |  | 3.76 (3.53, 4.03) |  |  |
| Marital status |  |  | ﹣0.85 ^a^ | 0.397 |  | ﹣0.52 ^a^ | 0.602 |
| Single | 54 (51.9) | 2.82 (2.65, 3.12) |  |  | 3.47 (3.13, 3.76) |  |  |
| Married | 50 (48.1) | 2.94 (2.60, 3.22) |  |  | 3.56 (3.25, 3.87) |  |  |
| Religion |  |  | 1.04 ^b^ | 0.791 |  | 0.24 ^b^ | 0.971 |
| Christian | 10 (9.6) | 2.71 (2.56, 3.13) |  |  | 3.38 (3.25, 3.82) |  |  |
| Buddhism | 14 (13.5) | 3.09 (2.60, 3.22) |  |  | 3.50 (3.09, 3.85) |  |  |
| Catholic | 11 (10.6) | 2.94 (2.59, 3.15) |  |  | 3.47 (3.18, 3.76) |  |  |
| None | 69 (66.3) | 2.82 (2.65, 3.12) |  |  | 3.53 (3.24, 3.82) |  |  |
| Education |  |  | 3.54 ^b^ | 0.171 |  | 1.34 ^b^ | 0.511 |
| Associate degree(3-year college) | 12 (11.5) | 2.82 (2.57, 2.94) |  |  | 3.32 (3.18, 3.53) |  |  |
| Bachelor’s degree | 70 (67.3) | 2.79 (2.65, 3.16) |  |  | 3.59 (3.19, 3.87) |  |  |
| ≥Master’s degree | 22 (21.2) | 3.06 (2.76, 3.22) |  |  | 3.47 (3.29, 3.81) |  |  |
| Total clinical experience (year) |  |  | 6.71 ^b^ | 0.035 |  | 1.21 ^b^ | 0.545 |
| < 5 | 26 (25.0) | 2.76 (2.54, 2.99) |  |  | 3.56 (3.18, 3.88) |  |  |
| 5–10 | 44 (42.3) | 2.97 (2.71, 3.19) |  |  | 3.53 (3.29, 3.82) |  |  |
| ＞10 | 34 (32.7) | 2.71 (2.53, 3.18) |  |  | 3.41 (3.13, 3.74) |  |  |
| Department |  |  |  |  |  |  |  |
| ICU | 36 (34.6) | 2.94 (2.63, 3.12) | 5.64 ^b^ | 0.130 | 3.53 (3.12, 3.82) | 0.01 ^b^ | 1.000 |
| Emergency department | 23 (22.1) | 3.06 (2.68, 3.35) |  |  | 3.47 (3.24, 3.91) |  |  |
| Hospice ward | 15 (14.4) | 2.76 (2.68, 3.15) |  |  | 3.59 (3.32, 3.74) |  |  |
| Cancer ward | 30 (28.8) | 2.74 (2.53, 2.99) |  |  | 3.56 (3.24, 3.76) |  |  |
| Current department experience (year) |  |  |  |  |  |  |  |
| < 5 | 55 (52.9) | 2.82 (2.59, 3.12) | 1.07 ^b^ | 0.586 | 3.53 (3.18, 3.82) | 0.63 ^b^ | 0.732 |
| 5–10 | 39 (37.5) | 2.94 (2.65, 3.18) |  |  | 3.65 (3.29, 3.82) |  |  |
| 11–15 | 10 (9.6) | 2.82 (2.60, 3.32) |  |  | 3.41 (3.24, 3.74) |  |  |
| Experience of death in the past year |  |  |  |  |  |  |  |
| No | 79 (76.0) | 2.82 (2.59, 3.12) | ﹣1.78 ^a^ | 0.075 | 3.53 (3.21, 3.82) | ﹣0.28 ^a^ | 0.781 |
| Yes | 25 (24.0) | 3.06 (2.71, 3.24) |  |  | 3.59 (3.18, 3.88) |  |  |
| Experience caring for patients with LST |  |  |  |  |  |  |  |
| No | 8 (7.7) | 2.71 (2.62, 2.85) | ﹣1.14 ^a^ | 0.256 | 3.47 (3.28, 3.63) | ﹣0.47 ^a^ | 0.638 |
| Yes | 96 (92.3) | 2.94 (2.63, 3.18) |  |  | 3.53 (3.18, 3.88) |  |  |
| Experience caring for patients with LST within the past year |  |  |  |  |  |  |  |
| No | 21 (20.2) | 2.94 (2.65, 3.18) | ﹣0.66 ^a^ | 0.509 | 3.41 (3.29, 3.59) | ﹣1.07 ^a^ | 0.283 |
| Yes | 83 (79.8) | 2.88 (2.59, 3.15) |  |  | 3.53 (3.18, 3.88) |  |  |
| Experience caring for patients with WLST |  |  |  |  |  |  |  |
| No | 3 (2.9) | 3.06 (3.03, 3.12) | ﹣1.13 ^a^ | 0.259 | 3.94 (3.71, 3.97) | ﹣1.32 ^a^ | 0.186 |
| Yes | 101 (97.1) | 2.82 (2.59, 3.18) |  |  | 3.53 (3.18, 3.82) |  |  |
| Experience caring for patients with WLST within the past year |  |  |  |  |  |  |  |
| No | 10 (9.6) | 3.09 (2.87, 3.40) | ﹣1.55 ^a^ | 0.122 | 3.47 (3.32, 3.93) | ﹣0.80 ^a^ | 0.424 |
| Yes | 94 (90.4) | 2.82 (2.60, 3.16) |  |  | 3.53 (3.18, 3.81) |  |  |
| Education related to LST within the past year |  |  |  |  |  |  |  |
| Never | 57 (54.8) | 2.94 (2.59, 3.18) | 0.31 ^b^ | 0.857 | 3.47 (3.24, 3.76) | 0.29 ^b^ | 0.866 |
| Once | 39 (37.5) | 2.82 (2.65, 3.12) |  |  | 3.65 (3.09, 3.91) |  |  |
| Twice or more | 8 (7.7) | 2.68 (2.60, 3.22) |  |  | 3.65 (3.24, 3.90) |  |  |
| Thoughts on a good death |  |  |  |  |  |  |  |
| Always (100 %) | 8 (7.7) | 2.97 (2.75, 3.25) |  |  | 3.85 (3.63, 4.04) |  |  |
| Often (80 %) | 22 (21.2) | 2.94 (2.71, 3.22) | 4.21 ^b^ | 0.378 | 3.74 (3.29, 3.94) | 5.77 ^b^ | 0.217 |
| Sometimes (50 %) | 45 (43.3) | 2.76 (2.59, 3.12) |  |  | 3.47 (3.18, 3.71) |  |  |
| Rarely (30 %) | 22 (21.2) | 2.82 (2.56, 2.94) |  |  | 3.53 (3.19, 3.76) |  |  |
| Never (0 %) | 7 (6.7) | 3.12 (2.68, 3.38) |  |  | 3.24 (2.94, 3.85) |  |  |

*Note*: Values are presented as *n* (%) or as *Median* (*P*_25_, *P*_75_) of the mean item scores, rather than summed total scores. The possible score ranges were 1–4 for good death recognition and 1–5 for attitude toward LST. ^a^ Mann–Whitney U test; ^b^ Kruskal–Wallis *H* test. WLST = Withdrawal of Life sustaining treatment. LST = Life sustaining treatment.

Appendix B. Participants’ characteristics (*n* = 8)

| Participant No. | Gender | Age  (years) | Professional role | Education level | Year of experience | Current unit |
| --- | --- | --- | --- | --- | --- | --- |
| 1 | Female | 27 | Nurse | Bachelor’s degree | 4 | Hospice |
| 2 | Male | 29 | Nurse | Bachelor’s degree | 4 | ED |
| 3 | Female | 30 | Nurse | Bachelor’s degree | 7 | ED |
| 4 | Male | 31 | Nurse | Bachelor’s degree | 6 | ICU |
| 5 | Male | 30 | Nurse | Bachelor’s degree | 5 | ED |
| 6 | Female | 30 | Nurse | Bachelor’s degree | 7 | ED |
| 7 | Female | 34 | Nurse | Master’s degree | 8 | ICU |
| 8 | Male | 36 | Nurse | Master’s degree | 11 | ICU |

*Note*: ED = Emergency Department.

 Appendix C. Concept of good death measure characteristics

| Subscale | Item content | *Mean* ± *SD* |
| --- | --- | --- |
| Clinical | 1. That it be painless or largely pain-free | 3.56 ± 0.54 |
|  | 2. That the dying period be short | 2.63 ± 0.83 |
|  | 3. That it be sudden and unexpected | 1.53 ± 0.84 |
|  | 5. That it occur naturally, without technical equipment | 2.69 ± 0.79 |
|  | 14. That death occurs during sleep | 2.85 ± 0.89 |
|  | Mean item score | 2.65 ± 0.44 |
| Closure | 4. That family and doctors follow the person’s wishes | 3.18 ± 0.76 |
|  | 6. That it be peaceful | 3.25 ± 0.71 |
|  | 7. That loved ones be present | 3.56 ± 0.62 |
|  | 8. That the person’s spiritual needs be met | 3.20 ± 0.76 |
|  | 9. That the person be able to accept death | 3.49 ± 0.64 |
|  | 10. That the person had a chance to complete important tasks | 3.40 ± 0.66 |
|  | 11. That the person had an opportunity to say good-bye | 3.63 ± 0.58 |
|  | 12. That the person was able to remain at home | 2.43 ± 0.77 |
|  | 13. That the person lived until a key event | 2.41 ± 0.78 |
|  | Mean item score | 3.17 ± 0.44 |
| Personal control | 15. That there be mental alertness until the end | 2.27 ± 0.80 |
|  | 16. That there be control of bodily function until death | 2.60 ± 0.88 |
|  | 17. That the ability to communicate be present until death | 2.71 ± 0.77 |
|  | Mean item score | 2.53 ± 0.70 |
| Mean item score | | 2.91 ± 0.39 |

Appendix D. Attitude toward life sustaining treatment

| Questions | | *Mean* ± *SD* |
| --- | --- | --- |
| 1 | Even for a LST patient, life should be extended using every available treatment methods.† | 3.54 ± 1.12 |
| 2 | If a LST patient and family request WLST due to financial difficulties, it should be allowed. | 3.57 ± 0.86 |
| 3 | If a patient choose WLST instead of pursuing painful treatment, it can be a way for the patient. | 4.11 ± 0.72 |
| 4 | WLST can be one of the methods for older patients with incurable diseases to end their life. | 3.84 ± 0.96 |
| 5 | If a LST patient or family request WLST due to religious faith, it should be respected. | 3.83 ± 0.85 |
| 6 | If a LST patient or family refuse airway intubation, it should be discontinued even if it is needed. | 3.48 ± 1.01 |
| 7 | If a LST patient or family request, pressor-agents should be stopped even if blood pressure is low. | 3.62 ± 0.94 |
| 8 | CPR for cardiac arrest should be performed for a patient on LST.† | 3.00 ± 1.43 |
| 9 | A patient’s family has a right to choose WLST on behalf of the patient. | 3.34 ± 0.87 |
| 10 | A patient has a right to choose WLST. | 4.67 ± 0.51 |
| 11 | WLST should be allowed for organ transplant. | 2.21 ± 0.97 |
| 12 | Use of ventilator for unconscious patient on LST should be discontinued if the pt's family request | 3.65 ± 0.83 |
| 13 | Weaning ventilator according to the family’s request is an act of consideration for the patient. | 3.38 ± 1.01 |
| 14 | Medical staff's decision of WLST is advisable if a patient with incurable status have no supporting family. | 3.30 ± 1.09 |
| 15 | A LST patient should be discharged under family's signature if the family want. | 3.47 ± 0.99 |
| 16 | It is advisable to get a consent on CPR prohibition when cardiac arrest is expected for the LST patient | 3.70±0.90 |
| 17 | It is unacceptable for health professionals just to watch patient dying without providing any treatments.† | 3.12 ± 1.17 |
| Mean item score | | 3.52 ± 0.48 |

*Note*:†Reverse score. WLST=Withdrawal of Life sustaining treatment. LST=Life sustaining

Appendix E. Influencing factors on attitude toward life sustaining treatment

| Variables | *B* | *SE* | *β* | *t* | *P* | 95 %*CI* |
| --- | --- | --- | --- | --- | --- | --- |
| Constant | 3.36 | 0.39 | - | 8.69 | <0.001 | 2.59, 4.13 |
| Good death recognition | 0.32 | 0.11 | 0.26 | 2.89 | 0.005 | 0.10, 0.54 |
| Gender(Male) | 0.80 | 0.22 | 0.32 | 3.55 | <0.001 | 0.36, 1.24 |

*Note:* Adjusted *R*^2^ = 0.16, *F* = 10.44, *P* < 0.001, Durbin-Watson’s *d* = 2.328
