# Supplementary material for: Attitudes of nurses toward life-sustaining treatment and its influencing factors: A mixed-methods study
Source: Int J Nurs Sci. 2026 Aug 14;13(5):459–66. doi: 10.1016/j.ijnss.2026.08.008 (PMC13648181; doi:10.1016/j.ijnss.2026.08.008)
Supplement: Supplementary file 2 [file mmc2.docx]

韩国护士对生命维持治疗的态度现状及影响因素分析：混合方法研究

Hyebin Oh, Juyoung Ha

**【摘要】**

**目的** 调查韩国护士对生命维持治疗的态度现状，并分析护士的善终认知与情境因素对该态度的影响。

**方法** 采用解释性顺序混合研究方法，于2024年7月至9月在韩国2所三级医院开展。定量阶段：选取104名护士，采用善终概念量表和生命维持治疗态度量表对其进行调查；采用多元线性回归分析识别与生命维持治疗态度相关的因素。在定性阶段：采用目的性抽样法，选取8名护士参与了半结构化访谈，并通过传统内容分析法对数据进行分析。研究结果通过联合展示的方式整合呈现。

**结果** 定量研究结果表明，护士对善终感知总均分为(2.91 ± 0.39)分,处于中等偏高水平，对生命维持治疗态度量表总均分为（3.52 ± 0.48）分。多元回归分析显示，男性（*β* = 0.32，*P* < 0.001）及较高的善终感知得分（*β* = 0.26，*P* = 0.005）与消极的生命维持治疗态度相关。定性研究提炼出7个主题：亲历亲友离世、直接的患者护理体验、临终关怀知识的深度、患者的年龄、预后和康复潜力、家庭经济负担、传统韩国文化价值观，以及社会接受度和法律责任方面的顾虑。综合分析显示，传统文化价值观和性别角色在解释性别关联方面起作用，而死亡经历及临终照护经验则解释了良好善终感知与生命维持治疗态度之间的关联。

**结论** 护士生命维持治疗态度受到良好善终感知及个人、临床和社会文化因素的影响。研究结果为护理管理者制订以能力为基础的临终关怀教育、标准化案例式诊疗方案和文化敏感的实践指南提供了实证依据，以支持护士在临终决策中的工作。

【**关键词**】死亡态度；生命维持治疗；护士；韩国；临终关怀

通信作者：Juyoung Ha, E-mail: [jyha1028@pusan.ac.kr](mailto:jyha1028@pusan.ac.kr)
